# Robust Glycogene-Based Prognostic Signature for Proficient Mismatch Repair Colorectal Adenocarcinoma

**DOI:** 10.3389/fonc.2021.727752

**Published:** 2021-10-07

**Authors:** Yixi Li, Dehua Li, Yang Chen, Yongping Lu, Fangbin Zhou, Chunhong Li, Zhipeng Zeng, Wanxia Cai, Liewen Lin, Qiang Li, Mingjun Ye, Jingjing Dong, Lianghong Yin, Donge Tang, Gong Zhang, Yong Dai

**Affiliations:** ^1^ Clinical Medical Research Center, The Second Clinical Medical College of Jinan University, Shenzhen People’s Hospital, Jinan University, Shenzhen, China; ^2^ Institute of Nephrology and Blood Purification, The First Affiliated Hospital of Jinan University, Jinan University, Guangzhou, China; ^3^ Key Laboratory of Functional Protein Research of Guangdong Higher Education Institutes and MOE Key Laboratory of Tumor Molecular Biology, Institute of Life and Health Engineering, Jinan University, Guangzhou, China; ^4^ Department of Nephrology, Dongguan Hospital of Guangzhou University of Traditional Chinese Medicine, Dongguan, China; ^5^ Guangxi Key Laboratory of Metabolic Diseases Research, Affiliated No. 924 Hospital, Southern Medical University, Guilin, China

**Keywords:** colorectal cancer, mismatch repair, glycosylation, glycogene, ceRNA, biomarker

## Abstract

**Background:**

Proficient mismatch repair (pMMR) colorectal adenocarcinoma (CRAC) metastasizes to a greater extent than MMR-deficient CRAC. Prognostic biomarkers are preferred in clinical practice. However, traditional biomarkers screened directly from sequencing are often not robust and thus cannot be confidently utilized.

**Methods:**

To circumvent the drawbacks of blind screening, we established a new strategy to identify prognostic biomarkers in the conserved and specific oncogenic pathway and its regulatory RNA network. We performed RNA sequencing (RNA-seq) for messenger RNA (mRNA) and noncoding RNA in six pMMR CRAC patients and constructed a glycosylation-related RNA regulatory network. Biomarkers were selected based on the network and their correlation with the clinicopathologic information and were validated in multiple centers (n = 775).

**Results:**

We constructed a competing endogenous RNA (ceRNA) regulatory network using RNA-seq. Genes associated with glycosylation pathways were embedded within this scale-free network. Moreover, we further developed and validated a seven-glycogene prognosis signature, GlycoSig (*B3GNT6*, *GALNT3*, *GALNT8*, *ALG8*, *STT3B*, *SRD5A3*, and *ALG6*) that prognosticate poor-prognostic subtype for pMMR CRAC patients. This biomarker set was validated in multicenter datasets, demonstrating its robustness and wide applicability. We constructed a simple-to-use nomogram that integrated the risk score of GlycoSig and clinicopathological features of pMMR CRAC patients.

**Conclusions:**

The seven-glycogene signature served as a novel and robust prognostic biomarker set for pMMR CRAC, highlighting the role of a dysregulated glycosylation network in poor prognosis.

## Introduction

Colorectal cancer (CRC) is the second leading cause of cancer-related deaths worldwide (1). Currently, the 5-year survival rate for patients with CRC varies from over 90% in stage I to slightly greater than 10% in stage IV (2). Colorectal adenocarcinoma (CRAC) is the most common pathohistological type of CRC. DNA mismatch repair (MMR) helps maintain DNA replication fidelity (3) and is therefore a well-established biomarker for CRC. MMR-proficient (pMMR) presents in 85% of CRAC (4). The prognostic outcome of pMMR patients is worse than that of MMR-deficient (dMMR) patients, and pMMR CRAC is more likely to metastasize (5).

Numerous studies have attempted to identify prognostic biomarkers. A commonly used method is to compare macromolecules in tumor and normal tissues and to identify differentially expressed proteins or RNAs. The currently reported prognostic biomarkers for CRC include proteins HOXB5 (6), VSTM2A (7), and STYX (8); long noncoding RNAs (lncRNAs) MCHR2, AC011472.4, and AC063944.1 (9); circular RNAs (circRNAs) circFADS2 (10), circ_0026344 (11), and hsa_circ_0004831 (12); and microRNAs (miRNAs) miR-1290 (13) and five-miRNAs set (14). However, different studies have reported different biomarkers probably due to the cohort selection, genetic background, treatment, and methodological differences within each study, which present significant system errors. Consequently, no prognostic biomarkers (including biomarker sets) have been commercialized as standardized diagnostic kits in clinical practice. Due to genome instability, heterogeneity, and complexity of cancer progression, biomarkers derived from blind screening of differentially expressed genes might not be a robust method for prognosis.

Although individual genes in tumors can mutate quickly, oncogenic pathways are much more conserved. Inspired by this fact, we changed the strategy of biomarker identification by identifying biomarkers from common oncogenic pathways of pMMR CRAC. For example, glycosylation is a key posttranslational modification that regulates single sugar moieties for targeted molecules, leading to glycan elongation (15). Tumor-associated glycosylation changes stem from a series of molecular causes, as glycan biosynthesis is a non-template-driven process (16). The genes involved in their biosynthesis, degradation, transport, and recognition are known as glycogenes (17). Notably, alterations in glycogenes have been shown to be associated with pMMR CRAC. Galectin-3 is often overexpressed in pMMR tissues compared to dMMR tissues (18). The Tn antigen is frequently upregulated in dMMR CRC compared with pMMR (19). The lack of GALNT6 was observed in more than 50% of dMMR tumors, but it was less frequent (<12%) in pMMR tumors (20). Downregulation of GALNT6 occurs during the transition from precancerous neoplasia to invasive carcinoma in a certain subset of tumors that frequently exhibit dMMR (21). Therefore, we aimed to discover prognostic biomarkers of pMMR CRAC based on the glycogene network.

## Materials and Methods

### Patients and Samples

**Figure 1:**
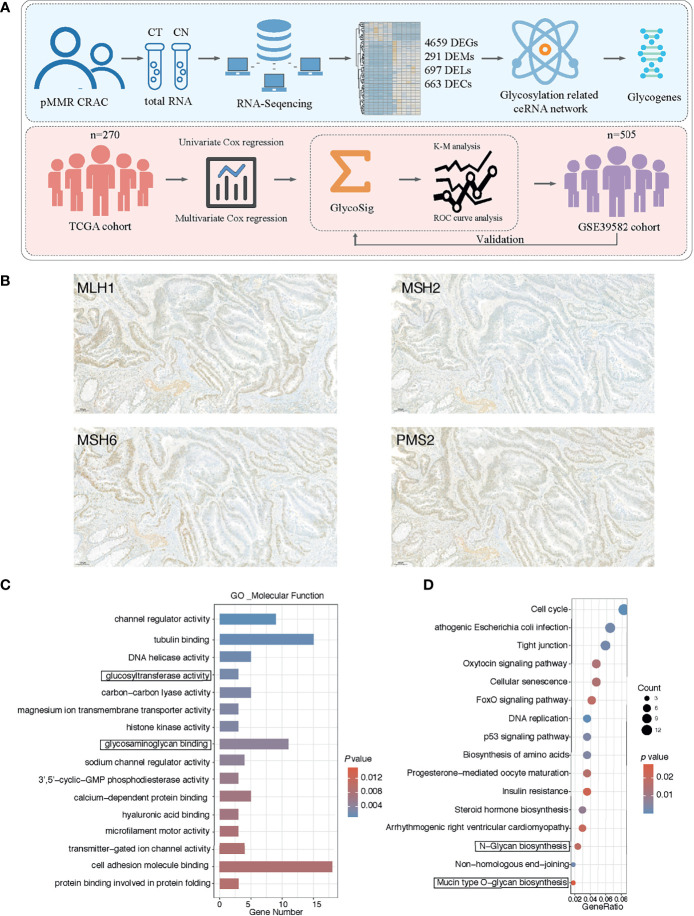
Cancer-specific messenger RNAs (mRNAs) in proficient mismatch repair (pMMR) colorectal adenocarcinoma (CRAC) and functional enrichment analysis. **(A)** Workflow of the experiment process. **(B)** The representative pattern of mismatch repair (MMR) status for CRAC tissues. **(C)** Top 16 enriched Gene Ontology (GO) terms of molecular function; y axis represents GO terms, and x axis represents gene number. Color of the bars represent enrichment significance. **(D)** Top 16 enriched Kyoto Encyclopedia of Genes and Genomes (KEGG) pathways; y axis represents pathway names, and x axis represents rich factor. Size and color of the bubble represent number of differentially expressed mRNAs (DEGs) enriched in the pathway and enrichment significance, respectively.

A total of six pMMR CRAC tissue and paired adjacent noncancerous tissue specimens were collected from patients who underwent operations in Shenzhen People’s Hospital (Shenzhen, China), and their clinical information is shown in **Table 1**. The new diagnosis of pMMR CRAC was confirmed by surgery and histopathologic examination, and patients had not received radiotherapy, chemotherapy, or biological immunotherapy before recruitment. The paired adjacent noncancerous tissue was defined as tissue that was at a 2.0-cm distance from the edge of the tumor and was free of tumor cells based on an experienced pathologist’s evaluation. All resected tissue samples were snap-frozen in liquid nitrogen and stored at -80°C until use. The study was approved by the Medical Ethics Committee of Shenzhen People’s Hospital, and consent was obtained from all participants. The workflow of the experimental process is illustrated in **Figure 1A**.

**Table 1:** Patients and tumor characteristics of the sequencing set.

Case No.	Gender	Age	Primary tumor location	TNM 8th	Stage	MMR status
1	M	55	Rectum	pT3/N1/M0	IIIB	pMMR
2	F	63	Left colon	pT3/N0/M0	IIA	pMMR
3	F	44	Left colon	pT3/N2a/M0	IIIB	pMMR
4	F	62	Right colon	pT2/N0/M0	I	pMMR
5	F	59	Rectum	pT3/N0/M0	IIA	pMMR
6	F	55	Rectum	pT4b/N1/M0	IIIC	pMMR

MMR, mismatch repair; pMMR, proficient mismatch repair.

### Total RNA Extraction and Quality Testing

Total RNA was extracted from pMMR CRAC tissues and paired non-tumorous tissues using TRIzol reagent (Invitrogen, USA). The RNA purity of each sample was quantified using a NanoDrop 2000 spectrophotometer (Thermo Fisher, USA). Total RNA quantity and integrity were assessed using a Bioanalyzer 2100 and RNA 6000 Nano LabChip Kit (Agilent, USA) with RNA integrity number (RIN) >7.0.

### Construction of the Small RNA Library and Sequencing

Approximately 1 µg of total RNA was used to prepare a small RNA library according to the TruSeq Small RNA Sample Prep Kit (Illumina, USA). Then, we performed single-end sequencing (50 bp) on an Illumina Hiseq 2500 (LC Sciences, China) following the manufacturer’s protocol.

### Read Alignment for miRNAs and Analysis of Differentially Expressed miRNAs

Raw data were analyzed as previously published (22). Raw reads were subjected to ACGT101-miR (LC Sciences, China) to remove adapter dimers, junk, low complexity, common RNA families, and repeats. Subsequently, unique sequences with lengths of ~18–26 nucleotides were mapped in miRBase 22.0 by BLAST search to identify known miRNAs and novel miRNAs. Differentially expressed miRNAs (DEMs) were selected with |log2 FC| ≥1 and p < 0.05 using the R package Ballgown.

### Construction of the Ribo-Zero RNA Library and Sequencing

Ribosomal RNA was removed from approximately 5 µg of total RNA using the Ribo-Zero Gold rRNA Removal Kit (Illumina, USA). After purification, the remaining RNA was fragmented (Magnesium RNA Fragmentation Module, NEB, USA) into small pieces using magnesium ions at 94°C. The cleaved RNA fragments were reverse-transcribed to create the final cDNA library in accordance with the protocols for commercial Illumina library preparation kits (Illumina, USA) (23). For reverse transcription, random primers were applied to the rRNA-depleted RNA to synthesize first-stranded RNA. Next, we synthesized U-labeled second-stranded DNAs using *Escherichia coli* DNA polymerase I (NEB, USA), RNase H (NEB, USA), and dUTP solution (Thermo Fisher, USA). An A-base was then added to the blunt ends of each strand, preparing them for ligation to the indexed adapters. Each adapter contained a T-base overhang to ligate the adapter to the A-tailed fragmented DNA. Single- or dual-index adapters were ligated to the fragments, and size selection (300–600 bp) was performed using AMPureXP beads. After heat-labile UDG enzyme (NEB, USA) treatment of the U-labeled second-stranded DNAs, the ligated products were amplified by PCR under the following conditions: initial denaturation at 95°C for 3 min; eight cycles of denaturation at 98°C for 15 s, annealing at 60°C for 15 s, extension at 72°C for 30 s; and a final extension at 72°C for 5 min. The average insert size of the final cDNA library was 300 ± 50 bp. Finally, we performed 2 × 150 bp paired-end sequencing on an Illumina Hiseq 4000 (LC-Bio Technology, China) following the vendor’s recommended protocol. Ribo-Zero RNA sequencing was used to analyze not only messenger RNAs (mRNAs) but also lncRNAs and circRNAs.

### Transcript Assembly and Analysis of Differentially Expressed mRNAs

Cutadapt (24) (https://cutadapt.readthedocs.io/en/stable) was used to remove reads containing adaptor contamination, low-quality bases, and undetermined bases. Sequence quality was verified using FastQC (25) (http://www.bioinformatics.babraham.ac.uk/projects/fastqc). We used Hisat2 (26) (https://ccb.jhu.edu/software/hisat2) to map reads to the genome of *Homo sapiens*. The mapped reads of each sample were assembled using StringTie (27) (http://ccb.jhu.edu/software/stringtie). Then, all transcriptomes from the samples were merged to reconstruct a comprehensive transcriptome using Gffcompare (https://github.com/gpertea/gffcompare). After the final transcriptome was generated, StringTie was used to determine mRNA expression levels by calculating FPKM (28). The differentially expressed mRNAs (DEGs) were selected with |log2 FC| ≥1 and p < 0.05 using the R package Ballgown. Driver genes for the DEGs were identified by using OncoVar analysis (29), and the result was shown in **Table S1**.

### LncRNA Identification and Analysis for Differentially Expressed lncRNAs

First, StringTie assembled and quantified the reads mapped to the genome of *Homo sapiens*. Second, transcripts that overlapped with known mRNAs and transcripts shorter than 200 bp were discarded. This is because lncRNAs are usually defined as RNAs that are >200 nt in length and do not encode proteins. We then utilized CPC (30) (http://cpc2.cbi.pku.edu.cn) and CNCI (31) (http://wwww.bioinfo.org/software/cnci) to predict transcripts with coding potential. All transcripts with CPC score <-1 and CNCI score <0 were retained without coding potential, and the remaining transcripts were considered lncRNAs. Differentially expressed lncRNAs (DELs) were selected with |log2 FC| ≥1 and p < 0.05 using the R package Ballgown.

### CircRNA Identification and Analysis for Differentially Expressed circRNAs

Cutadapt (24) and FastQC (25) were used as previously described. We first used TopHat2 (32) to map reads to the genome of *Homo sapiens*, which were removed as linear RNAs. Unmapped reads in the first step were mapped to the genome using the Tophat-fusion algorithm according to the splicing site features, such as GU/AG, GC/AG, and AU/AC. Back-spliced reads were identified in unmapped reads using the Tophat-fusion algorithm and CIRCExplorer (33). All samples were processed separately to identify circRNAs. circRNA expression from different samples was calculated using scripts in the house. Differentially expressed circRNAs (DECs) were selected with |log2 FC | ≥1 and p < 0.05 using the R package Ballgown.

### The Cancer Genome Atlas and Gene Expression Omnibus Data Mining

Gene expression and clinical information of COAD and READ patients from The Cancer Genome Atlas (TCGA) datasets were downloaded from UCSC xena (http://xenabrowser.net/), and the GSE39582 data were downloaded from Gene Expression Omnibus (GEO) (http://www.ncbi.nlm.nih.gov/geo/). The clinical data of the two cohorts are shown in **Table 2**. We selected all eligible CRAC samples with MMR status of the pMMR. First, each candidate gene was evaluated using univariate Cox regression analysis. Only candidate genes that correlated with prognosis were used for subsequent analysis. Then, all the significant genes in the univariate Cox regression were used to construct the prognostic GlycoSig model by multivariate Cox regression analysis. Kaplan–Meier (K-M) survival analysis and receiver operating characteristic (ROC) curve analysis were used to assess the predictive value of this GlycoSig model. The R packages on above workflow include “survival”, “survminer”, “GEOquery”, “ggplot2”, “Hmisc”, “grid”, “lattice”, “Formula”, and “rms.”

**Table 2:** Clinicopathologic characteristics of the different sets for patients with colorectal cancer.

Characteristics	COAD Cohort (n = 551)	READ Cohort (n = 186)	GSE39582 Cohort (n = 585)
**Mean age (SD, range), years**	67 (13, 31–90)	64 (12, 31–90)	67 (13, 54–80)
**Gender (male/female) (percent)**	284/262 (52/48)	99/87 (53/47)	323/263 (55/45)
**Pathological stage (percent)**			
I	86 (16)	35 (19)	37 (6)
II	219 (40)	55 (30)	266 (46)
III	152 (27)	56 (30)	209 (36)
IV	78 (14)	28 (15)	60 (10)
NA	16 (3)	11 (6)	13 (2)
**Primary tumor location (percent)**			
Rectum	0	186 (100)	NA
Left colon	218 (40)	0	NA
Right colon	309 (56)	0	NA
NA	24 (4)	0	NA
**Mismatch repair protein (percent)**			
pMMR	343 (62)	142 (76)	459 (79)
dMMR	66 (12)	9 (5)	77 (13)
NA	142 (26)	35 (19)	49 (8)
**Median follow-up (SD, range), months**	28 (26, 0–150)	24 (23, 0–131)	2 (1.3, 0–6.7)

dMMR, mismatch repair-deficient; MMR, mismatch repair; pMMR, proficient mismatch repair.

NA, Not Available.

### Functional Enrichment

Enrichment of Gene Ontology (GO) analysis: DEGs were classified by GO annotation into three categories: biological process, cellular component, and molecular function. For each category, a two-tailed Fisher’s exact test was used to test the enrichment of DEGs against all the identified mRNAs. GO with a corrected p < 0.05 was considered significant. For the Kyoto Encyclopedia of Gene and Genomes (KEGG) pathway enrichment analysis, survival analysis for all DEGs was first performed using the K-M algorithm combined with TCGA datasets. The enriched pathways were then tested for prognosis-related DEGs using the clusterProfiler R package. The pathway with a corrected p < 0.05 was considered significant. These analyses could identify the most enriched pathways in the DEGs. The most reliable biomarkers would be among them.

### ceRNA Network Construction and Network Attribute

Salmena et al. (34) proposed the competing endogenous RNA (ceRNA) hypothesis. This hypothesis states that all types of RNA transcripts communicate through miRNA response elements (MREs). The ceRNA network for glycosylation was constructed using the following steps: first, DELs, DECs, and 3ʹ-untranslated region (UTR) sequences of DEGs were predicted as miRNA targets using Miranda (Miranda Energy <-10) and TargetScan (TargetScan score ≥50) tools. Only the associations of miRNA–mRNA, miRNA–lncRNA, and miRNA–circRNA presented in the two methods were used to construct the ceRNA network. We then selected all genes related to glycosylation in the KEGG pathway enrichment analysis. DEM nodes related to glycogenes combined with DEL and DEC nodes were chosen to construct a sub-ceRNA network. Finally, the ceRNA network was generated using Cytoscape 3.8.2. The mRNAs and miRNAs with p < 0.05, |log2 FC| ≥1 and lncRNAs and circRNAs with p < 0.01 and |log2 FC| ≥3 were retained in the ceRNA network. To identify the nature of the network, we used the poweRlaw package to fit the power law distribution of the node degree distribution and created a power law scatter plot.

### miRNA-Related Heatmap

We calculated the degree of all nodes in the ceRNA network using Cytoscape and then selected the top 10 degrees of miRNAs and the mRNAs related to these miRNAs. Finally, we plotted a heatmap to show these molecules using the pheatmap R package.

### Statistical Analysis

The paired-samples t-test was used for the significance analysis of DEGs, DELs, DECs, and DEMs. The likelihood ratio test was used for multiple Cox regression analysis and univariate Cox regression analysis. The log-rank test was used for K-M survival analysis. Statistical analyses were carried out using SPSS Statistics 19.0 (SPSS Inc., USA). Other analyses were carried out by R version 3.5.2 with the following packages: “edgeR”, “pheatmap”, “forestplot”, “rms”, “ggplot2”, “survivalROC”, and “survival”. All hypothesis testing was two-sided, and p < 0.05 was defined as statistically significant.

## Results

### Glycogenes Are Enriched in Differentially Expressed mRNAs

We collected the tumor and normal tissues of six pMMR CRAC. The positive MMR status of all tumor tissues was confirmed using immunohistochemistry against the MMR proteins MLH1, MSH2, MSH6, and PMS2 (**Figure 1B**). We performed mRNA sequencing (mRNA-seq) for paired tumors and normal tissues. At the threshold of |log2 FC| ≥1 and p < 0.05, a total of 4,659 DEGs were identified in the pMMR CRAC tissues of the six patients studied, compared with adjacent non-tumor tissues, including 2,783 upregulated DEGs and 1,876 downregulated DEGs (**Figure S1A**). *MSH2* as one of the driver genes was identified by using OncoVar analysis (**Table S1**), indicating the significant role of MMR status for CRC patients. GO and KEGG pathway enrichment analysis showed that the glycosylation-related pathways were among the top 3 enriched GO molecular function terms (**Figure 1C**). The two most enriched terms were tubulin binding (35) and cell adhesion molecule binding (36), which are universal for cancers as previously reported. Moreover, the glycan synthesis pathways were also among the most enriched KEGG pathways (**Figure 1D**). These results showed that glycogenes are commonly dysregulated in the pMMR CRAC.

### The miRNA–lncRNA–circRNA–mRNA ceRNA Network of Glycogenes

**Figure 2:**
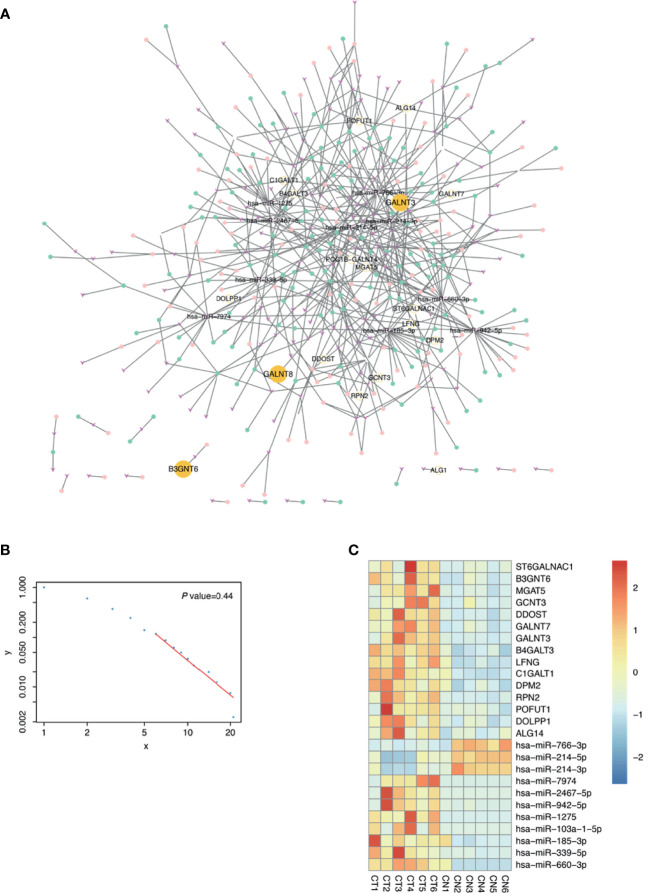
Construction of glycosylation related competing endogenous RNA (ceRNA) network. **(A)** The ceRNA network related to glycosylation. Fuchsia v shapes represent microRNA (miRNA); green dots, long noncoding RNA (lncRNA); pink dots, circular RNA (circRNA); yellow dots, messenger RNA (mRNA). **(B)** Power law scatter plot for ceRNA network. **(C)** Top 10 mRNA–miRNA interactions of ceRNA network in proficient mismatch repair (pMMR) colorectal adenocarcinoma (CRAC) tissues (CT1-6) and non-tumor tissues (CN1-6) were visualized in heatmap.

As numerous glycogenes were differentially expressed in the RNA-seq and considering the potential bias and errors of omics technology, identifying true signatures of pMMR CRAC requires further mining of these data. The disorder of ceRNA crosstalk profoundly contributes to the CRC process (37). Glycogenes interact with and are regulated by the miRNA–lncRNA–circRNA ceRNA network and are thus stabilized in tumor cells. As for textbook knowledge, regulatory networks are more conserved than individual genes. Therefore, the DEGs that are highly robust in the regulatory network were more likely to be signatures. To construct such a network, we sequenced the miRNAs, lncRNAs, and circRNAs of the six patients. At the threshold of |log2 FC| ≥1 and p < 0.05, a total of 291 DEMs, 697 DELs, and 663 DECs were identified in pMMR CRAC tissues compared with adjacent non-tumor tissues (**Figures S1B–D**). Using such criteria to identify nodes and edges, a ceRNA network for glycosylation pathways consisting of 18 DEGs, 152 DEMs, 153 DELs, and 115 DECs was constructed (**Figure 2A**), and the heatmap for DEGs, DEMs, DELs, and DECs is shown in **Figures S3A, D–F**, respectively. Most of the nodes are interconnected as a main graph, and the degree of the nodes follows the power law distribution (**Figure 2B**), suggesting that they form a typical biological scale-free network. This indicates that the glycogenes tend to be tightly connected by various noncoding RNAs. The top 10 mRNA–miRNA interactions in the ceRNA network showed 15 upregulated glycogenes and corresponding miRNAs (**Figure 2C**). Notably, these DEGs and DEMs were clearly valid in all six samples, indicating the robustness of the network-based screening strategy.

### GlycoSig Model for the Prognosis of Proficient Mismatch Repair Colorectal Adenocarcinoma

**Figure 3:**
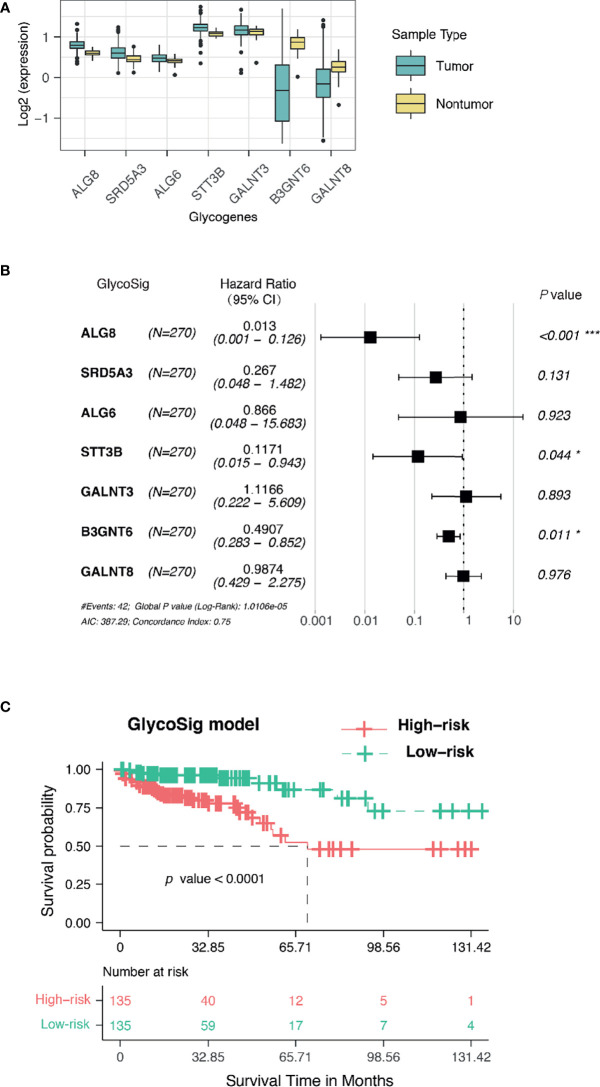
GlycoSig model for the prognosis of proficient mismatch repair (pMMR) colorectal adenocarcinoma (CRAC) in The Cancer Genome Atlas (TCGA) dataset. **(A)** The expression of seven glycogenes in tumor and non-tumor tissues from TCGA database. **(B)** Forest plots showed the multivariate Cox regression analysis for GlycoSig; *P < 0.05; ***P < 0.001. **(C)** Kaplan–Meier survival curves of pMMR CRAC patients classified into high- and low-risk groups using the GlycoSig.

Assuming that aberrant glycosylation is a hallmark of CRAC, we further investigated prognosis-related glycogenes for pMMR CRAC. We first performed univariate Cox regression analysis for all candidate genes related to glycosylation in KEGG pathway to evaluate the correlation between gene expression and overall survival (OS) through TCGA database for pMMR CRAC patients (n = 270). Seven glycogenes were statistically significant and thus considered variables for prognosis (**Table 3**), including five upregulated genes and two downregulated genes for TCGA dataset (**Figure 3A**). *B3GNT6*, *GALNT3*, and *GALNT8* belong to O-linked glycosylation processes, while *ALG8*, *STT3B*, *SRD5A3*, and *ALG6* belong to N-linked glycosylation processes. The seven-glycogene expression heatmap of in-house samples is shown in **Figure S3C**. Finally, we found that a signature based on glycogenes (GlycoSig) could be a prognostic factor for pMMR CRAC patients using multivariate Cox regression analysis (p < 0.0001; **Figure 3B**). Regression coefficients were calculated for the entire set, and the risk score of GlycoSig was identified for each patient using the following formula:


GlycoSig risk score=(−4.4×ALG8)+(−1.3×SRD5A3)+(−0.14×ALG6)+(−2.1×STT3B)+(0.11×GALNT3)+(−0.71×B3GNT6)+(−0.013×GALNT8)

**Table 3:** Univariate Cox regression analysis for clinical features and candidate genes.

		Univariate analysis			
		HR	95% CI		*P* value
**Clinical features**	Sex	1.30	0.69	2.40	0.421
	Age less than 65 years	2.60	1.20	5.40	0.012*
	Primary tumor location				
	Rectum	1.30	0.53	3.00	0.598
	Right colon	1.40	0.64	2.90	0.426
	Pathologic stage				
	Stage I	0.11	0.02	0.59	0.010*
	Stage II	0.24	0.08	0.76	0.015*
	Stage III	0.56	0.19	1.70	0.301
	Stage IV	0.39	0.10	1.60	0.189
**Candidate genes**	*ALG8*	0.017	0.0017	0.17	<0.0001***
	*SRD5A3*	0.19	0.04	0.92	0.040*
	*ALG6*	0.03	0.0023	0.39	0.007**
	*STT3B*	0.07	0.0095	0.52	0.009**
	*GALNT3*	0.21	0.058	0.75	0.016*
	*B3GNT6*	0.48	0.32	0.72	<0.001***
	*GALNT8*	0.47	0.27	0.82	0.008**

HR, hazard ratio.

*P < 0.05, **P < 0.01, ***P < 0.001.

In particular, *B3GNT6*, *GALNT3*, and *GALNT8* as members of GlycoSig were also included in the ceRNA network, suggesting that this biomarker set should be robust. *B3GNT6* [hazard ratio (HR) = 0.491, 95% CI 0.283–0.852, p = 0.011], *ALG8* (HR = 0.013, 95% CI 0.001–0.126, p<0.001), and *STT3B* (HR = 0.117, 95% CI 0.015–0.943, p = 0.044) might be associated with the development of poor prognosis of pMMR CRAC. The 270 patients were categorized into high- and low-risk groups using the median risk score of GlycoSig as the cutoff. Survival analysis was performed using the K-M method with a log-rank statistical test. Patients in the high-risk group had significantly worse OS than those in the low-risk group (median OS = 5.7 years, log-rank p < 0.0001; **Figure 3C**).

### The Prognostic Power of the GlycoSig Biomarker Set

**Figure 4:**
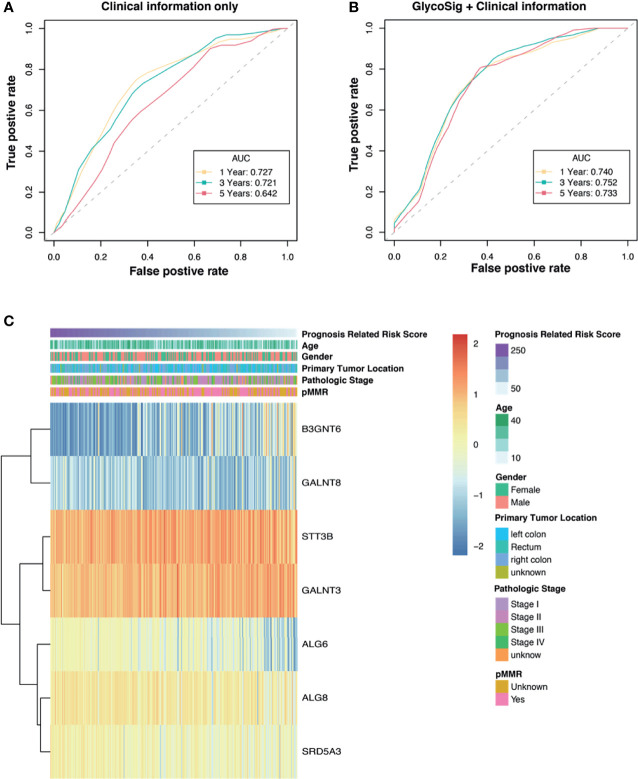
The prognostic power of the GlycoSig biomarker set. Receiver operating characteristic (ROC) curve analysis of clinicopathologic information **(A)** and GlycoSig combined with clinical information **(B)**; Yellow, green, and red dotted lines represent GlycoSig risk score predicting 1-, 3-, and 5-year overall survival. **(C)** Heatmap of the GlycoSig combined with clinical features and messenger RNA (mRNA) expression of glycogenes in The Cancer Genome Atlas (TCGA) dataset.

Clinicopathological factors of patients with pMMR CRAC are also known to have prognostic value. To compare the GlycoSig biomarker set to such clinicopathological factors, we performed univariate Cox regression analysis for clinicopathological factors, including age, sex, primary tumor location, and pathological stage, on TCGA data of 270 patients. Age less than 65 years (HR = 2.6, 95% CI 1.2–5.4, p = 0.012), stage I (HR = 0.11, 95% CI 0.02–0.59, p = 0.010), and stage II (HR = 0.24, 95% CI 0.077–0.76, p = 0.015) were significantly correlated with OS of pMMR CRAC patients (**Table 3**). The clinical information alone reached the areas under the ROC curve (AUC) of 0.727, 0.721, and 0.642 at 1, 3, and 5 years, respectively (**Figure 4A**). For the GlycoSig model only, the AUC reached 0.720, 0.741, and 0.714 at 1, 3, and 5 years, respectively (**Figure S2H**), indicating that the performance of GlycoSig was robust. Combining the clinical information with GlycoSig, the AUC reached 0.740, 0.752, and 0.733 at 1, 3, and 5 years, respectively (**Figure 4B**). The elevation of AUC, especially the remarkable increase in the long term, showed the prognostic power and robustness of the GlycoSig biomarker set. When the patients were sorted according to the GlycoSig risk score, their clinical features were randomly distributed. As for the seven glycogenes in GlycoSig, *B3GNT6*, *ALG6*, *ALG8*, and *SRD5A3* showed a slight correlation with the risk score, while the other three genes were visibly random (**Figure 4C**). In fact, these gene expression levels fluctuate considerably in general. The expression of single glycogenes could also provide prognostic value to a certain extent (**Figures S2A–G**), but not as significant as the biomarker set. These results also validated the necessity of the multi-gene GlycoSig in terms of sensitivity and robustness.

### GlycoSig Validation and Nomogram Constructed

**Figure 5:**
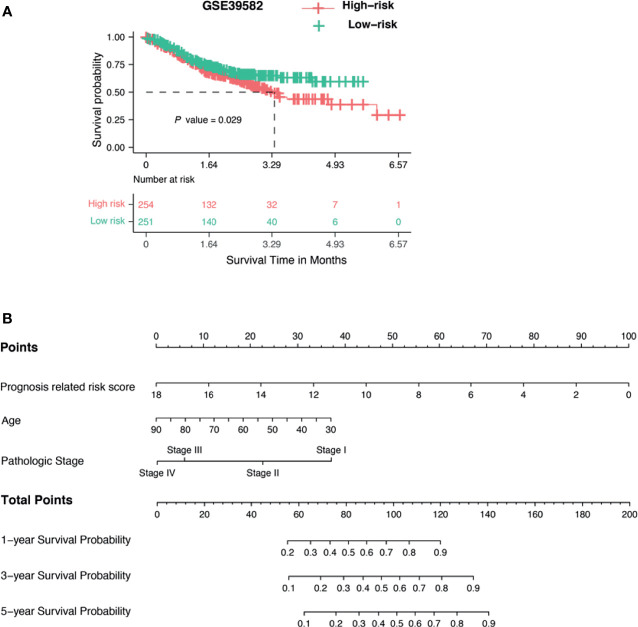
Validation of GlycoSig and nomogram for proficient mismatch repair (pMMR) colorectal adenocarcinoma (CRAC). **(A)** Independent validation of GlycoSig in the GSE39582 set; Kaplan–Meier survival curves of patients classified into high- and low-risk groups using the GlycoSig for overall survival. **(B)** Nomograms to predict 1-, 3-, and 5-year survival probability in pMMR CRAC.

Many biomarkers can only be used in restricted cohorts and are not robust when tested in different populations. To test whether our GlycoSig is widely applicable, we downloaded data from 505 patients from the GEO database (accession number: GSE39582) (38), including the transcriptome profiles and clinical features. The patients in GSE39582 were from the French National Cartes d’Identité des Tumeurs (CIT) program. The risk scores were calculated for each patient. Interestingly, the survival analysis showed that patients with a high-risk score for GlycoSig had a shorter survival time than those with a low-risk score (p = 0.029; **Figure 5A**). This validated the robustness of the GlycoSig biomarker set and its general applicability. We further constructed a simple-to-use nomogram that integrated the risk score of GlycoSig and clinicopathological features to predict the 1-, 3-, and 5‐year survival probabilities of patients who had undergone surgical resection (**Figure 5B**). The points from each independent prognostic factor listed in the nomogram were summated. The total points were calculated by adding up the corresponding points of each individual covariate on the point scale.

## Discussion

CRAC is heterogeneous and characterized by different molecular and phenotypic characteristics (39). Although MMR testing can provide important information for clinical decision-making in CRAC, pMMR CRAC patients with the same clinical features may have different prognostic and therapeutic responses. Previous research on prognostic biomarkers has generally found differentially expressed genes using next-generation sequencing or proteome mass spectrometry. However, this strategy does not produce robust biomarker sets generally due to cohort selection. A multicenter study is a possible way to avoid center-originated bias. However, methodological bias makes the normalization of multicenter datasets very difficult. Here, our strategy is to avoid blind screening by focusing on specific pathways and constructing the RNA regulatory network, which is more conserved than individual genes. We focused on aberrant glycosylation, which is a hallmark of CRC and has been shown to be altered during tumor development and progression (40). Indeed, our strategy found a signature of seven glycogenes based on the functional pathway and ceRNA network.

In particular, aberrant glycosylation has been confirmed to protect cancer cells from radiotherapeutic and drug-targeted treatments. Ionizing radiation treatment for CRC increases the sialylation of β1-integrin, which enhances CRC cell adhesion, migration, and invasion (41). The curative effect of anti-vascular endothelial growth factor (VEGF) therapy depends on glycosylation of the cancer cell surface, and the removal of α2-6-linked sialic acid results in tumor resistance to anti-VEGF therapy (42). It was noted that specific glycans in tumor cells, such as Tn antigen, could serve as a novel immune checkpoint, offering new immunotherapeutic opportunities (43). A novel recombinant human chimeric immunoglobulin G (IgG)1 anti-Tn antibody, named Remab6, represents a new useful reagent to detect Tn-positive glycoproteins as a biomarker for human carcinomas and may also be a novel therapeutic agent for targeted cancer treatment (44). In addition, the monoclonal antibody CH129 that targets tumor-associated sialylated glycan demonstrated its potential for multimodal cancer therapy (45). Hence, targeting aberrant glycosylation as an immunotherapeutic strategy is a major field of research against CRC.

Three GlycoSig genes, *B3GNT6*, *GALNT3*, and *GALNT8*, have been noticed by other studies in CRC. Iwai et al. (46) demonstrated that the expression of *B3GNT6* is significantly decreased in CRC tissues and is a useful marker for distinguishing benign adenomas from premalignant lesions. Moreover, decreased expression of *B3GNT6* is related to epithelial–mesenchymal transition (EMT) and metastasis in CRC (46–48), which provides evidence for the prognostic value of *B3GNT6*. In addition, *GALNT3* has been shown to be regulated by the linc01296/miR-26a network in CRC (49). Tang et al. (50) found that lncRNA *GAU1* regulates the expression of *GALNT8* in CRC. The overexpression of *GALNT8* significantly accelerates the cell cycle and thus promotes CRC cell lines and correlates with poor prognosis in CRC patients. Silencing of *GALNT8* suppresses the cancer cell proliferation and induced resistance against oxaliplatin in CRC cell lines. The results suggested that the *GALNT8* may play as a CRC prognosis marker and potential target against chemoresistance (50).

The other four genes of GlycoSig have not been investigated in the context of CRC; however, many studies have indicated their significance in other cancer types. Previous studies have shown that *ALG8* could be a variate of prognostic model for gastric adenocarcinoma (51) and frequently amplified hotspot on 11q14.1 (*ALG8*) associated with significantly worse prognosis for breast cancer (52). The *ALG6* single-nucleotide polymorphism (SNP) is a potential prognostic biomarker for cutaneous melanoma and is likely through modulating gene expression (53). Hsu et al. (54) demonstrate that EMT enriched programmed death-ligand 1 (PD-L1) in cancer stem-like cells by the EMT/β-catenin/STT3/PD-L1 signaling axis, in which EMT transcriptionally induced N-glycosyltransferase STT3 (including isoforms STT3A and STT3B) through β-catenin, and subsequent STT3-dependent PD-L1 N-glycosylation stabilized and upregulated PD-L1. Zhang et al. (55) found that *SRD5A3* was highly expressed in breast cancer tissues, and high *SRD5A3* expression was related to poorer prognosis. Also, Mai et al. (56) demonstrated that *SRD5A3* is upregulated in hepatocellular carcinoma (HCC) tissues, and higher *SRD5A3* level predicts poor OS, progression-free survival, relapse-free survival, and disease-specific survival in HCC patients. *SRD5A3* polymorphism may contribute to a genetic predisposition for prostate cancer (57). Metastatic prostate cancer expressed higher transcript levels for *SRD5A3 (* 58). The SRD5A3 enzyme converts testosterone to dihydrotestosterone (DHT). It is highly expressed in metastatic prostate cancer compared to benign and localized prostate cancer (59). Knockdown of *SRD5A3* expression in prostate cancer cells resulted in a significant decrease in DHT production and a drastic reduction in cell viability (60). These findings indicate that *SRD5A3* should be a promising molecular target for prostate cancer therapy.

Although most of the glycogenes are not well characterized in pMMR CRAC patients, they showed mechanistic and diagnostic significance in other cancers. As a textbook knowledge, pathways are more conserved than single proteins. Therefore, such information also indicates the potential mechanistic contribution of these glycogenes in CRAC. As we provided robust evidence for their connection to prognosis, further experimental studies on these prognosis-related glycogenes are worth conducting. Another limitation of this study is that the prognostic value of the GlysoSig was validated in two retrospective cohorts. Therefore, the applicability of the GlysoSig should be further verified in prospective pMMR CRAC cohorts. Also, their mechanism in pMMR CRAC is worth an investigation.

In conclusion, we screened and validated the seven-glycogene prognostic signature (GlycoSig) for pMMR CRAC patients using the pathway–network strategy. These findings indicate that the seven glycogenes should be a promising molecular target for pMMR CRAC therapy.

## Data Availability Statement

The raw data of RNA sequencing are available in the GSA for Human (https://bigd.big.ac.cn/gsub/) of BIG Sub at accession number HRA000868 and HRA000866. Other datasets generated during the current study are available from the corresponding authors upon reasonable request.

## Ethics Statement

The studies involving human participants were reviewed and approved by the Medical Ethics Committee of Shenzhen People’s Hospital. The patients/participants provided their written informed consent to participate in this study.

## Author Contributions

YD, DT, and LY designed the research. FZ, CL, ZZ, WC, and LL collected the data. YXL, DL, YC, and YPL performed the data analysis. YXL and GZ wrote the article (YXL drafted the initial article; GZ and YXL finalized the article). QL, MY, and JD critically reviewed the article. YD and DT had primary responsibility for the final content. All authors contributed to the article and approved the submitted version.

## Funding

This study was supported by the Science and Technology Plan of Shenzhen (JCYJ20200109144218597, JCYJ20190807145815129), Guangxi Key Laboratory of Metabolic Diseases Research (20-065-76), Guangzhou Science and Technology Program (201802030010), Guangdong Engineering Technology Research Center (507204531040), and Dongguan Social Science and Technology Development Project (201950715002195), and National Natural Science Funds of China (81802916).

## Conflict of Interest

The authors declare that the research was conducted in the absence of any commercial or financial relationships that could be construed as a potential conflict of interest.

## Publisher’s Note

All claims expressed in this article are solely those of the authors and do not necessarily represent those of their affiliated organizations, or those of the publisher, the editors and the reviewers. Any product that may be evaluated in this article, or claim that may be made by its manufacturer, is not guaranteed or endorsed by the publisher.
