# Supplementary material for: Robust Glycogene-Based Prognostic Signature for Proficient Mismatch Repair Colorectal Adenocarcinoma
Source: Front Oncol. 2021 Oct 7;11:727752. doi: 10.3389/fonc.2021.727752 (PMC8529276; doi:10.3389/fonc.2021.727752)
Supplement: Supplementary file 1 [file DataSheet_1.docx]

**Table S1: Driver genes from the DEGs were identified by using OncoVar analysis**

| TCGA Dataset | Gene Name | Log2FC | *P* Value | Regulation |
| --- | --- | --- | --- | --- |
| COAD | *QKI* | 1.82 | 5.40E-05 | down |
| COAD | *ERBB3* | 1.92 | 0.00030 | up |
| COAD | *SALL4* | 3.87 | 0.00031 | up |
| COAD | *RNF43* | 3.74 | 0.00051 | up |
| COAD | *RSPO3* | -1.96 | 0.00064 | down |
| COAD | *ERBB2* | 1.60 | 0.00064 | up |
| COAD | *SOX9* | 4.53 | 0.00074 | up |
| COAD | *CASP8* | 1.80 | 0.00096 | up |
| COAD | *PIK3R1* | 1.17 | 0.0015 | down |
| COAD | *TP53* | 1.80 | 0.0016 | up |
| COAD | *RSPO2* | 4.50 | 0.0020 | down |
| COAD | *MUTYH* | 1.16 | 0.00200 | up |
| COAD | *POLE* | 1.33 | 0.0023 | up |
| COAD | *NBEA* | 2.46 | 0.0044 | down |
| COAD | *CTNNB1* | 1.15 | 0.0046 | up |
| COAD | *SRC* | 1.39 | 0.0057 | up |
| COAD | *ELF3* | 3.16 | 0.0066 | up |
| COAD | *AXIN1* | 1.07 | 0.010 | up |
| COAD | *MSH2* | 1.06 | 0.012 | up |
| COAD | *PTPRT* | 1.25 | 0.028 | down |
| COAD | *EIF3E* | 1.02 | 0.038 | up |
| READ | *RUNX1T1* | 3.14 | 0.00030 | down |
| READ | *SOX9* | 4.53 | 0.00074 | up |
| READ | *PIK3R1* | 1.17 | 0.0015 | down |
| READ | *TP53* | 1.80 | 0.0016 | up |
| READ | *PTPRT* | 1.25 | 0.028 | down |


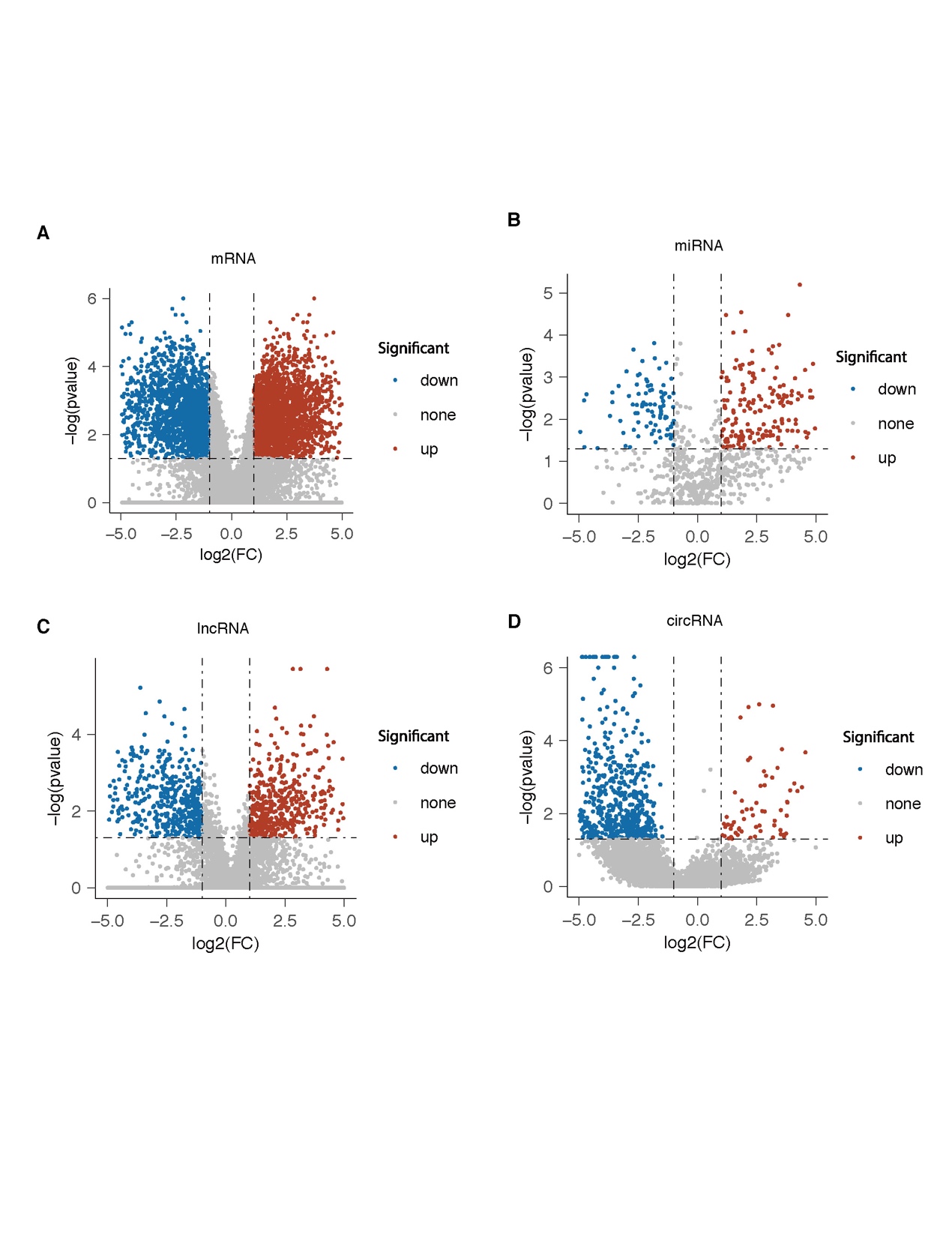


**Figure S1: Differential analysis for transcriptome data in pMMR CRAC**

Differentially expressed mRNAs (DEGs), miRNAs (DEMs), lncRNAs (DELs), and circRNAs (DECs) in pMMR CRAC tissues and paired adjacent nontumour tissues were visualized in volcano plots (**A**, **B**, **C**, and **D)**, respectively. Red and blue dots indicated upregulated and downregulated genes; gray dots presented for no significant difference.


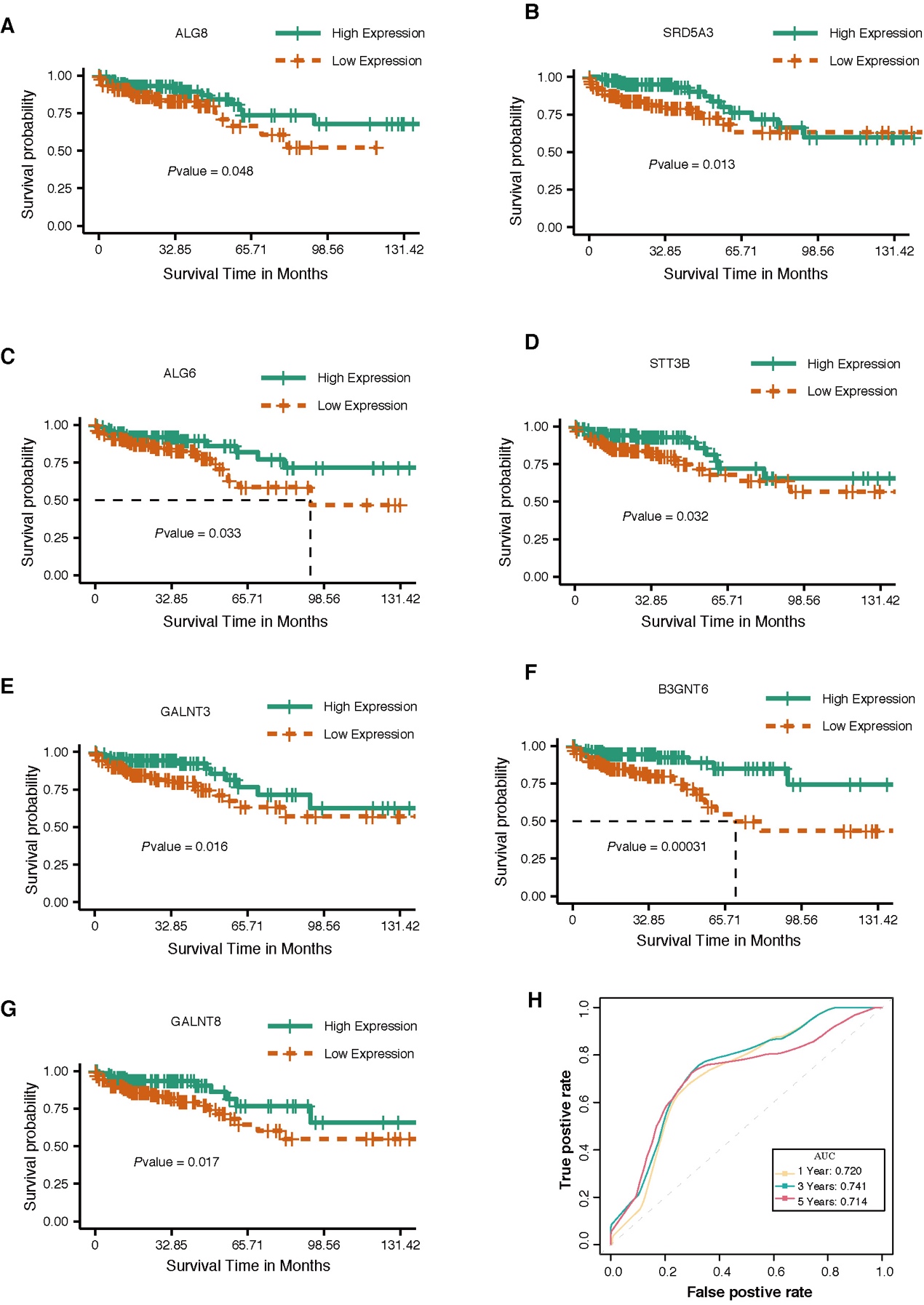


**Figure S2: The prognostic power of single glycogenes for pMMR CRAC**

Kaplan-Meier survival curves of pMMR CRAC patients (**A-G**) were classified into high and low-expression groups of single glycogenes from GlycoSig. The low expressin of glycogenes related to worse survival. (**H**) ROC curve analysis of GlycoSig only; Yellow, green and red dotted lines represent GlycoSig prognostic model for predicting 1, 3 and 5-year overall survival.


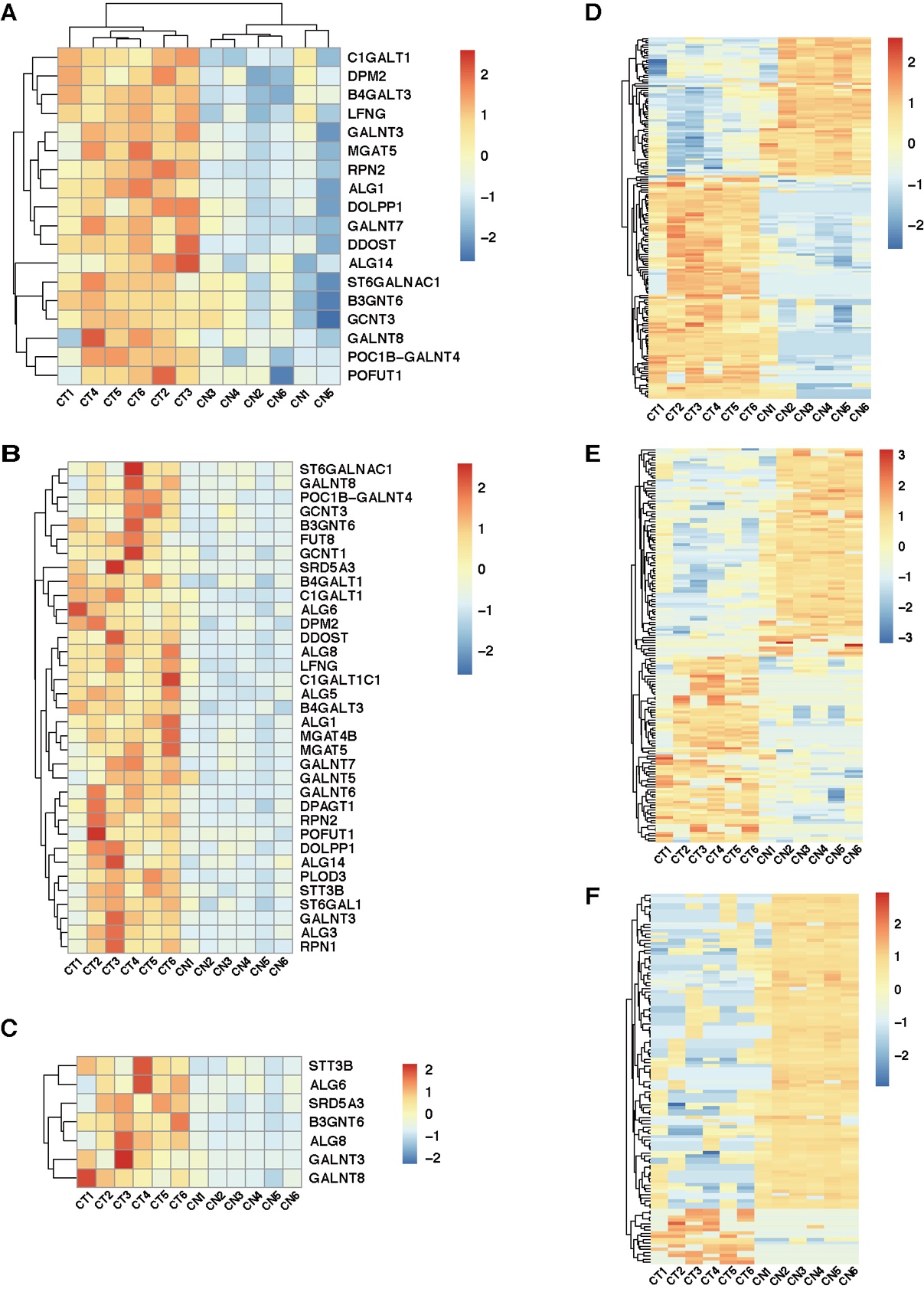


**Figure S3: The heatmap for the expression of RNAs of pMMR CRAC in-house**

The DEGs (**A**), DEMs (**D**), DELs(**E**) and DECs(**F)** in glycosylation related ceRNA network of pMMR CRAC tissues (CT1-6) and nontumour tissues (CN1-6) were visualized in heatmaps, respectively; (**B**) The DEGs of glycosylation pathways from KEGG enrichment analysis for in-house samples; (**C**) The DEGs expression of seven glycogenes from GlycoSig for in-house samples.
